# The Effects of Water Parameters on Monthly Seagrass Percentage Cover in Lawas, East Malaysia

**DOI:** 10.1155/2013/892746

**Published:** 2013-09-17

**Authors:** E. I. Ahmad-Kamil, R. Ramli, S. A. Jaaman, J. Bali, J. R. Al-Obaidi

**Affiliations:** ^1^Institute of Biological Sciences, University of Malaya, 50603 Kuala Lumpur, Malaysia; ^2^Malaysian Nature Society (MNS), JKR 641, Jalan Kelantan, Bukit Persekutuan, 50480 Kuala Lumpur, Malaysia; ^3^MOE-HICoE Marine Endangered Species (MES) Program, Institute of Oceanography & Environment (INOS), Universiti Malaysia Terengganu, 21030 Kuala Terengganu, Malaysia; ^4^Sarawak Forestry Corporation, Lot 218, KCLD, Jalan Tapang, Kota Sentosa, 93250 Kuching, Sarawak, Malaysia; ^5^Agro-biotechnology Institute (ABI), c/o MARDI Headquarters, 43400 Serdang, Selangor, Malaysia

## Abstract

Seagrass is a valuable marine ecosystem engineer. However, seagrass population is declining worldwide. The lack of seagrass research in Malaysia raises questions about the status of seagrasses in the country. The seagrasses in Lawas, which is part of the coral-mangrove-seagrass complex, have never been studied in detail. In this study, we examine whether monthly changes of seagrass population in Lawas occurred. Data on estimates of seagrass percentage cover and water physicochemical parameters (pH, turbidity, salinity, temperature, and dissolved oxygen) were measured at 84 sampling stations established within the study area from June 2009 to May 2010. Meteorological data such as total rainfall, air temperature, and Southern Oscillation Index were also investigated. Our results showed that (i) the monthly changes of seagrass percentage cover are significant, (ii) the changes correlated significantly with turbidity measurements, and (iii) weather changes affected the seagrass populations. Our study indicates seagrass percentage increased during the El-Nino period. These results suggest that natural disturbances such as weather changes affect seagrass populations. Evaluation of land usage and measurements of other water physicochemical parameters (such as heavy metal, pesticides, and nutrients) should be considered to assess the health of seagrass ecosystem at the study area.

## 1. Introduction

Seagrasses are marine flowering plants that have the ability to complete their life cycle while fully submerged in marine environment constraints [1]. These marine plants cover large geographic area worldwide [2]. Seagrasses provide services and goods for their ecological community, for example, wave protection, reduced water flow, fishing ground, and oxygen production [3–8]. Decline in seagrass species and coverage was observed worldwide [9, 10]. Combination of environmental changes such as physical parameters (temperature, salinity, and pH), natural phenomena (tidal effects, cyclone, and seasonal changes) and anthropogenic parameters has affected seagrass population [2, 11–16]. Other complex interactions such as water quality, grazers, and nutrients affect seagrass population by causing seagrass mortality [17].

The coastlines of Southeast Asia (SEA) are often exposed to heavy rain and tropical thunderstorms during wet season or monsoon. In Thailand, temporal interactions between seagrass percentage cover and species distribution of *Cymodocea rotundata *and* Thalassia hemprichii *during different seasons (dry and wet) have been observed [18]. Meanwhile, in Pulau Tinggi, Malaysia, seagrass leaf length of *Cymodocea serrulata, Halodule uninervis*, and *Syringodium isoetifolium *increased after the monsoon season [16]. In Malaysia, monsoon season, which occurred annually from November to February, is characterized by heavy rainfall coupled with thunderstorm and rough sea conditions which brings heavy waves upon reaching shallow water [19]. Similar weather conditions of the monsoon and its effects on seagrass populations were studied in other tropical areas such as Queensland, Australia. Here, temporal changes of seagrass cover after flood and cyclone related events have been observed [20, 21]. Seagrass population is devastated by these natural events due to uprooting of the plants, elevated turbidity, and seagrass seed burial [20]. In the event of seagrass burial and sediment removal, effect on seagrasses depends on the condition of the rhizome and root system of the seagrass species. For example, *Syringodium filiforme* which has delicate rhizome and root system was uprooted and buried, while the population of *Thalassia testudinum *which has robust rhizome and root system was unaffected by hurricane [22]. In temperate waters, seagrass losses resulted from physical damage as well as reduction in water quality caused by the cyclone [23, 24]. Seagrass coverage recovery after these events has been observed. Full recovery was observed after three years which was facilitated by improved water quality [21]. Meanwhile, in Florida, seagrasses were recovered in deeper area after two years of hurricane which were also dependent on improve water quality such as decrease of turbidity and increase of light availability [24].

Until recently, there has been no study on the monthly changes and the effects of water parameter fluctuations on the seagrass populations in Malaysia. Seagrass meadows are known to contribute indirectly to Malaysian fishery stock by providing nursery and nesting grounds to high number of fisheries species [3, 7, 8, 25]. Seagrass habitat in Malaysia is also frequented by protected marine wildlife such as the green turtles and dugongs [26, 27]. Therefore, the dynamics of seagrass population should be studied to address the declining population of seagrasses in Malaysia [14]. In this paper, we investigate (i) if seagrass percentage cover shows monthly variation, (ii) if monthly changes of water physicochemical parameters such as pH, temperature, turbidity, salinity, and dissolved oxygen are causing changes on monthly seagrass percentage cover, (iii) other events that might cause changes on monthly seagrass percentage cover. The results of the present study showed that seagrass population was observed to respond towards monthly variations of water physicochemical parameters. Weather changes, nutrients, and the physical characteristics of seagrasses were suggested as drivers that control seagrass population at the study area.

## 2. Material and Method

### 2.1. Study Site

**Figure 1:**
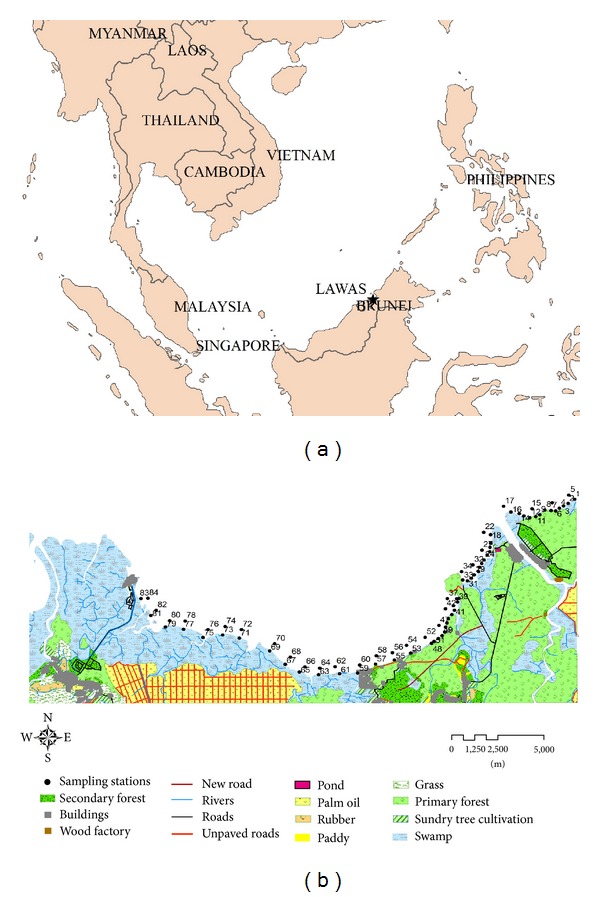
Map representing the location of the (a) study area in Lawas, Sarawak, East Malaysia and (b) the sampling stations established.

The study was conducted on an intertidal seagrass meadow along the coastline of Lawas (Figure 1), Sarawak, East Malaysia (4°59′3.5016′′N, 115°27′4.3992′′E). The beach profile of this coastal area includes rocky shores, sandy beach, mudflats, mangrove fringing forest, and seagrass beds. The coastline is greatly influenced by freshwater runoffs from nearby rivers. Estimation of seagrass percentage cover and water parameter measurements was conducted monthly at 84 sampling stations established along the 32.2 km of coastline from Sg. Bangkulit (4°59′3.5016′′N, 115°27′4.3992′′E) to Awat-awat (4°55′59.8002′′N, 115°14′37.899′′E) from June 2009 to May 2010.

### 2.2. Seagrass Sampling

Seagrass population was observed at each sampling station by wadding or snorkelling depending on the water depth. At each sampling station, three 50 cm × 50 cm quadrats were randomly thrown within 5 m radius of the sampling stations [28]. Total seagrass cover within the quadrat was visually estimated using the percent cover standards used by Seagrass-Watch [29]. Seagrass species within the quadrats were identified and the percent contribution of each species to the total coverage was determined. Data obtained from three quadrats at each sampling station were averaged.

### 2.3. Water Parameter Measurements

To test whether water physicochemical parameters are effecting monthly seagrass population, dissolved oxygen (mg/L), pH, temperature (°C), and salinity (PSU) were measured using HI9828 HANNA Instrument Multimeter and turbidity (FTU) was measured using HI93703 HANNA Instrument Microprocessor Turbidity meter at each sampling station during high tide to reduce the river influence on the measurements. These instruments were placed over the seagrass meadows and readings were taken three times. Due to the lack of baseline water parameter data on the study area and the need for values on water physicochemical parameters relevant to seagrass distribution, economical limitations, and logistic constraints, only these water physicochemical parameters were measured monthly for this study.

### 2.4. Meteorological Data

Rainfall data for Limbang station (4°48′N, 115°0′E) in Sarawak were obtained from Malaysian Meteorological Department. Sampling was conducted during El-Nino period [30]. The stage of El-Nino events can be determined based on Southern Oscillation Index (SOI), which were obtained from the Australian Government Bureau of Meteorology.

## 3. Data Analysis

Averages of seagrass percentage cover, salinity, temperature, pH, dissolved oxygen, and turbidity (mean ± SE) were plotted against the sampling monthly. The differences of monthly seagrass percentage cover were analyzed by grouping the stations based on B-B Scale, a scale modified from Braun-Blanquet Scale [31], and Participatory Coastal Resource Assessment (PCRA) Scale [32] (Table 1). The temporal changes of grouped seagrass percentage cover were analyzed using nonparametric statistical analysis, Friedman test. The Friedman test is a nonparametric repeated measures ANOVA. It is useful to test differences between several related groups when assumptions for repeated measures ANOVA are violated and when data is ordinal [33]. 

Relationship of monthly water physicochemical parameters with grouped seagrass percentage cover was evaluated using nonparametric Spearman Rho correlation.

## 4. Results

### 4.1. Seagrass Composition

**Figure 2:**
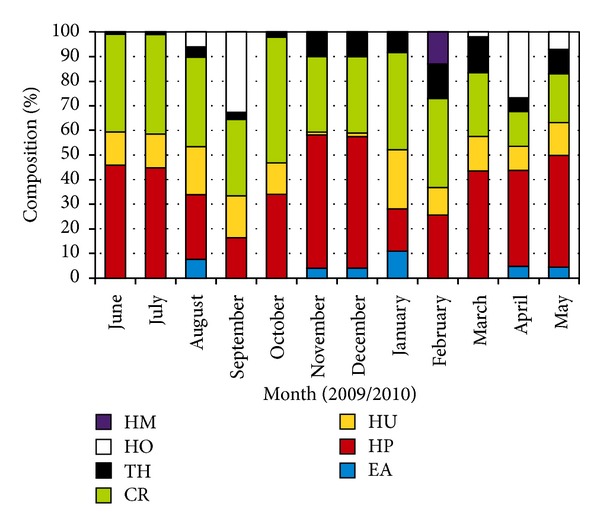
Monthly changes of mean seagrass species composition June 2009 to May 2010 (HM: *Halophila minor*, HO: *Halophila ovalis*, TH: *Thalassia hemprichii*, CR: *Cymodocea rotundata*, HU: *Halodule uninervis*, HP: *Halodule pinifolia*, and EA: *Enhalus acoroides*).

*Thalassia hemprichii*,* Cymodocea rotundata*,* Halodule pinifolia*,* Halodule uninervis*,* Enhalus acoroides*,* Halophila minor*,* Halophila ovalis*, and* Halophila beccarii* were found at coastal stations on sandy, muddy, mix of sand and mud, and mix of sand and coral rubble substrate at the intertidal zone which was exposed during low tide. Monthly seagrass composition shows that *Halodule pinifolia* is the most abundant seagrass species at the study area (Figure 2). 

### 4.2. Monthly Changes of Seagrass Percentage Cover

**Figure 3:**
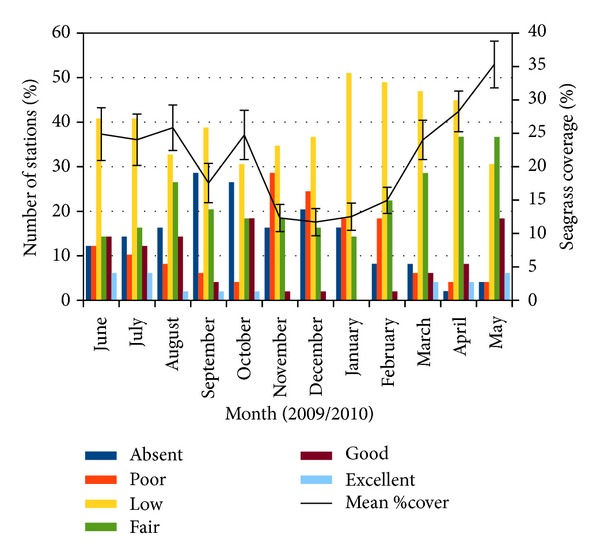
Monthly variations of grouped seagrass percentage cover and mean seagrass percentage cover (%) ± S.E from June 2009 to May 2010.

Average seagrass percentage cover varied from 11.7% to 35.2% with the highest value observed in May 2010 (Figure 3). Mean of seagrass percentage cover was observed to be stable from June to August 2009. Seagrass decline was observed in September 2009 but recovered in October 2009 but declined again in November 2009. Mean seagrass percentage cover recovered in February 2009 and reached its peak in May 2010. The fluctuations of seagrass percentage cover can be observed by the variation of the number of stations classified in a specific group scale each month. Monthly changes of grouped seagrass percentage cover differed significantly (*χ* ^2^_(11,*N*=49)_ = 88.260, *P* < 0.001). 

### 4.3. Meteorological Data

**Figure 4:**
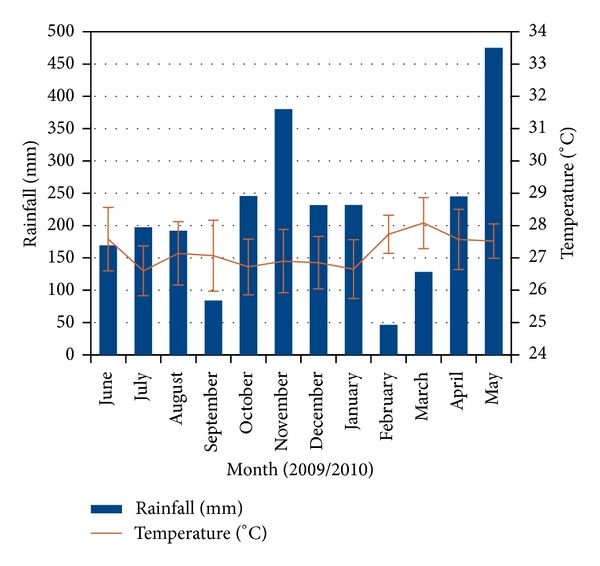
Total rainfall (mm) and air temperature (°C) ± S.E June 2009–May 2010.

**Figure 5:**
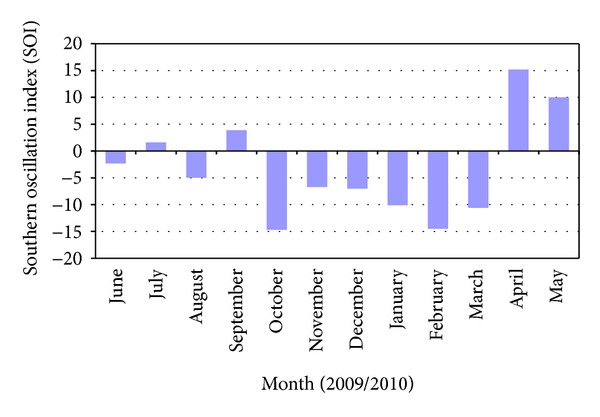
Southern Oscillation Index (SOI) from June 2009 to May 2010.

The total rainfall from June 2009 to May 2010 varied between 46.4 mm to 475 mm with the highest value observed to be 101 mm in October 29, 2009. In addition to these conditions, average air temperature varied between 26.6°C and 28.1°C with the highest value observed of 29.5°C in March 6, 2010 (Figure 4). Southern Oscillation Index (SOI) observed negative SOI values from October 2009 to March 2010 (Figure 5).

### 4.4. Correlation between Grouped Seagrass Percentage Cover and Basic Water Physicochemical Parameters

**Figure 6:**
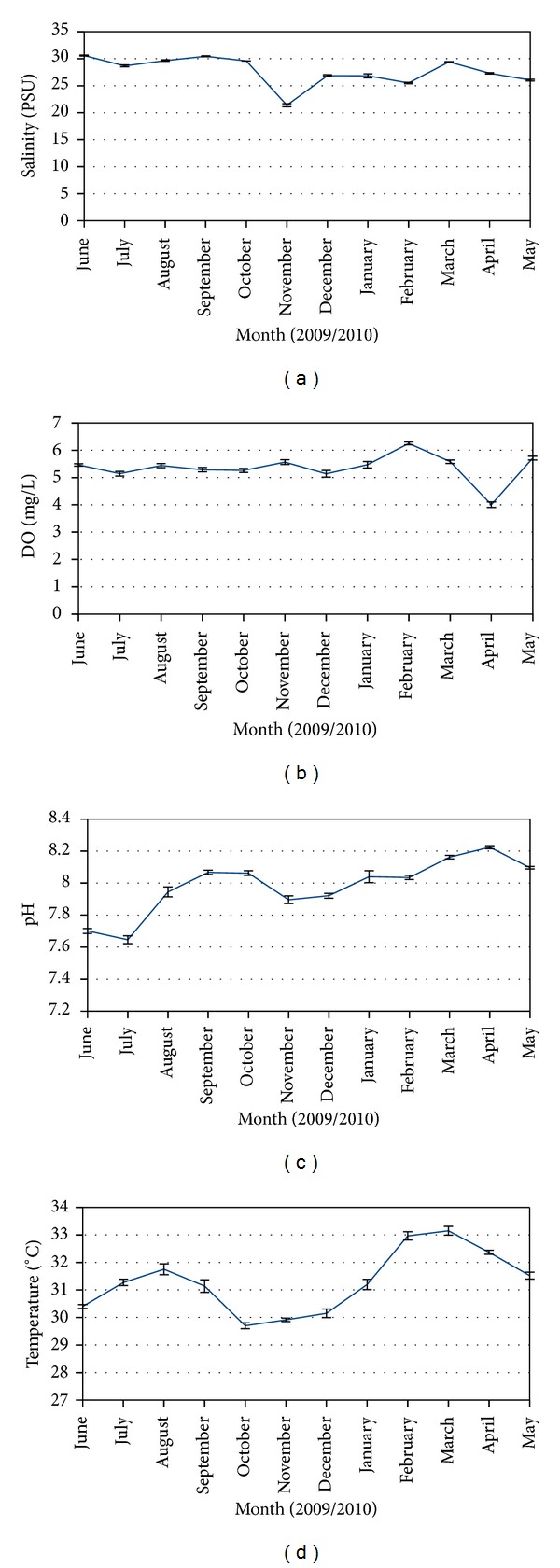
Monthly variations of (a) salinity (PSU) ± S.E, (b) dissolved oxygen (mg/L) ± S.E, (c) pH ± S.E, and (d) temperature (°C) ± S.E in Lawas.

**Figure 7:**
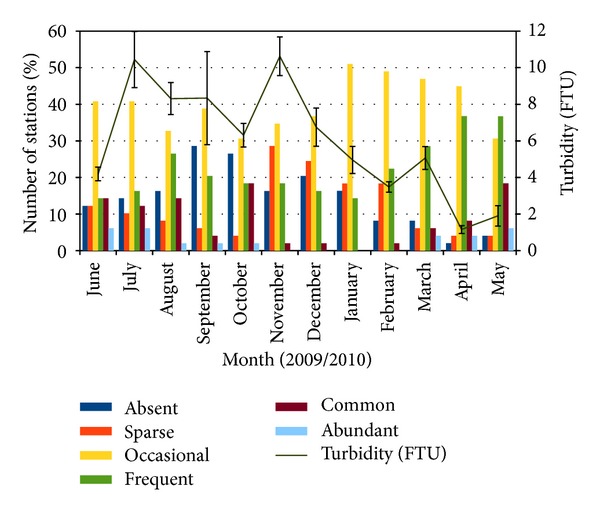
Monthly variations of grouped seagrass percentage cover and turbidity (FTU) ± S.E.

The current study provided a baseline data for basic water physicochemical parameters (water temperature, turbidity, pH, salinity, and dissolved oxygen) of the study area (Figure 6). Correlation analysis between monthly water physicochemical parameters and grouped seagrass percentage cover showed that only turbidity significantly correlated with grouped seagrass percentage cover (*r*_(588)_ = −0.030, *P* < 0.05, Figure 7). High turbidity measurements were recorded in July and November 2009. Two months after high turbidity event in July 2009, seagrass percentage cover decreased in September 2009. Higher turbidity event in November 2009 caused seagrass percentage cover to decrease in December 2009, with the number of stations classified as abundant, frequent, and common decreases. In January 2010 when lower turbidity was recorded (starting from January 2010 to May 2010), seagrass percentage cover improved (number of stations classified as abundant, frequent, and common increase). 

## 5. Discussion

Temporal changes of seagrass population are a normal phenomenon in a seagrass ecosystem due to weather changes, such as flood, hurricane, and cyclone [20–22]. However, as the environment is changing and responding towards human induced threats, the decline of seagrass population should be carefully investigated. The current study showed significant monthly changes of seagrass percentage cover. Mean seagrass percentage cover was observed to decline in September and November 2009. This result is similar to many previous studies which have observed decline on seagrass population. For instance, the decrease of above ground biomass of *Halodule uninervis* in Indonesia [34] and seagrass percentage cover and biomass of *Halodule uninervis*,* Thalassia hemprichii*, and *Halophila ovalis *declined in Pulau Gaya, Sabah [14]. Similar findings were recorded in Townsville, Australia, where percentage cover of *Halodule uninervis*,* Halodule *sp., and *Halophila ovalis* was also observed to be declining [35].

Turbidity has always been found to affect seagrasses [14, 36, 37]. The current study demonstrated that turbidity is responsible for causing seagrass decline in September 2009. Earlier, turbidity was observed to cause *Halophila ovalis *and *Halodule pinifolia *mortality [36, 37]. Increase of suspended material and light attenuation caused decline in shoot density of seagrasses at Cape Bolinao, Philippines [38], and productivity and abundance of *Posidonia oceanica* in Spain [17]. There are several possibilities which can explain the decline of mean seagrass percentage cover due to elevated turbidity. Elevated turbidity limits light from reaching the leaves for photosynthetic processes which will decrease seagrass productivity [17]. In addition, the decline of seagrass populations during increased turbidity event can also be explained by the shading of old shoots (resulting in reduced percentage cover) and formation of new shoots which will improve better light harvesting for photosynthesis [38]. 

During the monsoon season in November 2009, estimation of seagrass percentage cover indicated that there is a decline of mean seagrass percentage cover. Heavy rain increases the volume of freshwater runoff from the rivers which causes increased turbidity and sedimentation in coastal waters [23, 39]. Previous observations by [20, 21, 23] have recorded similar weather conditions which also caused decline on seagrass percentage cover. Seagrass populations were devastated by these natural events due to the uprooting of the plants, low light condition due to elevated turbidity, and seagrass burial [20].

Although the decline of seagrass population was observed, seagrass recovery is expected as seagrasses have the capability to recolonize disturbed meadow by the extension of its rhizomes and the presence of seagrass seed bank [40]. Seagrass population in Lawas was observed to recover immediately after the rainy season in February 2010. This finding is not surprising as seagrass in many parts of the world had recovered/recolonized after extreme weather conditions and disturbances [16, 20, 21, 24]. However, the result of this study showed increase in the seagrass percentage cover during the 2009/2010 El-Nino phenomenon. The finding of this study is contradictory with many previous findings. Seagrass percentage cover was found to be decreasing in subtropical area such as northern Australia during the El-Nino phenomenon [41, 42]. Results due to El-Nino that occurred between 1997 and 1998 caused more than 2,000 acres of seagrass in the southwest part of Florida [43] to develop. There are several possible answers to explain the recovery of seagrass observed in this study. (i) Additional nutrient from the land, which is brought to the sea during the rainy season, may contribute to the recovery of seagrass percentage cover [16]. Nutrients such as phosphate and nitrate increase growth and cover of *Thalassia hemprichii *[44] and *Halodule uninervis *[45]. (ii) The recovery of seagrass percentage cover took place during the drought season which is immediately after the rainy season ends. As the Southern Hemisphere experiences summer, dry winds from the South Pacific limit humidity in Borneo and cause few parts of Borneo including Lawas to experience several weeks of drought. Less rain limits the amount of freshwater input to the coastal area resulting in improved water clarity. In addition to this, El-Nino phenomenon in Malaysia is characterized by high air and sea water temperature which have effects directly on reducing cloud cover and limit total rainfall [30]. During this period, it could be possible that the amount of available light is sufficient for seagrass growth which leads to population recovery. (iii) Seagrass recovery in Lawas persisted until the end of the sampling period in May 2010 may be contributed by the nature of the fast growing *Halodule pinifolia* [28, 46] which was abundant at the study area.

More generally, our results show that weather events and turbidity have been observed to be the main driver that affects monthly seagrass percentage cover. Although the present study was successful in proving that seagrass percentage cover was influenced by water parameter measurements (i.e., turbidity), it is important to understand that the water physicochemical parameters were only measured during high tide. As a result, tidal effects on seagrass population can be studied if seagreass sampling and water physicochemical parameters measurements were studied during low tide. Moreover, the study was conducted with the assumption that time of water parameter does not influence the monthly measurements. Therefore, the result of this study may be regarded as providing a baseline data on basic water physicochemical parameters and the correlation with seagrass percentage cover at intertidal areas during high tide.

The current study used a classification modified from the Braun-Blanquet and PCRA scales to find differences of monthly seagrass percentage cover and its correlation with water physicochemical parameters. Braun-Blanquet score, which is widely used in seagrass monitoring, estimates seagrass density based on the number of seagrass shoots and assign a value from the scale from 0 to 0.5. However, the Braun-Blanquet scale was not suitable for the current study because this study does not take into account solitary shoots as <5% cover [31]. Meanwhile, PCRA developed a scale to assess the health of mangrove ecosystem based on crown cover, average tree height and measurements of regeneration. Instead of giving score, PCRA scale gives names to the condition of the mangrove as excellent, good, fair, and poor [32]. The current study was also unable to adopt PCRA scale because the PCRA scale gathered all plant covers from 0% to 25% as one classification. 

Seagrasses in Lawas are important nursery and breeding ground for juvenile fish species [7]. Small scale fish culture can be found along the main rivers of Lawas. Other than fisheries, the coastlines of Lawas are being exploited for agricultural purposes. Therefore, the evaluation of land-usage and measurements of other water parameters (such as heavy metal, pesticides, and nutrients) should be considered to assess the overall health of seagrass ecosystem at the study area. 

## Figures and Tables

**Table 1:** B-B scale.

Scale	Coverage (%)
Absent	No seagrass found in quadrat (0% cover)
Sparse	<5%
Occasional	5–25%
Frequent	26–50%
Common	51–75%
Abundant	76–100%
